# Challenging choice: a media study of anti-abortion movements in India

**DOI:** 10.1080/26410397.2026.2653891

**Published:** 2026-04-15

**Authors:** Ragini Bordoloi, Vinitha Jayaprakasan, Souvik Pyne, Alka Barua

**Affiliations:** a Abortion Theme Member, CommonHealth, Mumbai, India; b Abortion Theme Member, CommonHealth, New Delhi, India; c Chairperson and Abortion Theme Member, CommonHealth, New Delhi, India; d Abortion Theme Lead, CommonHealth, Ahmedabad, India. *Correspondence*: alki75@hotmail.com; alkibarua@gmail.com

**Keywords:** anti-abortion, anti-choice activism, media, abortion access, autonomy, reproductive rights, reproductive justice

## Abstract

In recent years, the backlash against abortion and reproductive autonomy has grown, driven by authoritarian regimes and conservative ideologies. In 2023, CommonHealth conducted a study to identify key anti-choice entities in India, examine their global links, and analyse their narratives and strategies for shaping public opinion and influencing policy through media platforms (print and social) in three languages. Key findings were that these organisations are leveraging educational platforms and social media, co-opting campaigns against gender-based discrimination, and framing their arguments with moral and constitutional rhetoric. Their tactics include the manipulation of public perception and gaining trust by blending science, religion, and emotional appeals. Crisis helplines, educational outreach, social media campaigns, mass mobilisation efforts, and direct peer interactions are utilised to particularly influence youth and garner support against reproductive choice. The study also highlights the growing influence of international networks on these initiatives. The emerging narratives are increasingly focused on intertwining anti-abortion and broader anti-choice ideologies, emphasising fetal rights and the sanctity of life. These initiatives thus pose a significant threat to reproductive rights and autonomy by undermining access to safe and legal abortion services. To counter this evolving anti-abortion activism, it is crucial to develop context-specific interventions and strategies. This analysis aims to contribute to a nuanced understanding of the challenges, calling for interdisciplinary collaboration among stakeholders to safeguard and strengthen reproductive justice. Reconceiving abortion as a life-enhancing decision is therefore essential to advancing reproductive rights and autonomy amidst escalating anti-choice narratives.

## Plain language summary

Abortion rights remain a divisive issue worldwide. The 2022 reversal of Roe v. Wade in the United States gave new energy to anti-abortion groups globally. Even in countries like India, where abortion laws are relatively liberal, the effects were still felt. Websites and social media posts in English, Hindi, and Assamese were tracked using targeted keywords. Desk research on activism in the US and UK added a global context. The study found eight key groups, including NGOs and faith-based organisations. Their messages highlight the fetus's rights from conception. They view abortion as immoral and illegal. They use educational platforms and social media. They spread medical misinformation and borrow words from gender equality campaigns. Their strategies include crisis helplines, media campaigns, public marches, and direct peer interactions. These efforts undermine access to safe, legal abortion. Our findings highlight the need to challenge these narratives and defend reproductive autonomy.

## Résumé

Ces dernières années, les réactions contraires à l’avortement et à l’autonomie reproductive se sont intensifiées, alimentées par les régimes autoritaires et les idéologies conservatrices. En 2023, CommonHealth a réalisé une étude en Inde pour identifier les principaux acteurs anti-choix, examiner leurs liens internationaux et analyser leurs discours et leurs stratégies de manipulation de l’opinion publique et d’influence des politiques dans les médias (presse écrite et réseaux sociaux) en trois langues. L’étude a révélé que ces organisations exploitent les plateformes éducatives et les réseaux sociaux, s’approprient les campagnes contre les discriminations sexistes et alignent leurs arguments sur une rhétorique morale et constitutionnelle. Leurs tactiques consistent notamment à manipuler la perception du public et à gagner sa confiance en mêlant science, religion et arguments émotionnels. Des lignes d’écoute téléphonique d’urgence, des actions de sensibilisation, des campagnes sur les réseaux sociaux, des activités de mobilisation de masse et des échanges directs entre pairs sont utilisés en particulier pour influencer les jeunes et mobiliser un soutien contre le choix en matière de procréation. L’étude souligne aussi l’influence croissante des réseaux internationaux sur ces initiatives. Les discours émergents tendent de plus en plus à mêler les idéologies anti-avortement et anti-choix plus générales, en insistant sur les droits du fœtus et le caractère sacré de la vie. Ces initiatives représentent donc une menace importante pour les droits et l’autonomie en matière de procréation, en entravant l’accès à des services d’avortement sûr et légal. Pour contrer cette montée de l’activisme anti-avortement, il est crucial de mettre au point des interventions et des stratégies adaptées au contexte. Cette analyse vise à contribuer à une compréhension nuancée des enjeux, en préconisant une collaboration interdisciplinaire entre les parties prenantes afin de protéger et de renforcer la justice reproductive. Repenser l’avortement comme une décision qui améliore la vie est donc essentiel pour faire progresser les droits et l’autonomie en matière de procréation face à la montée des discours anti-choix.

## Resumen

En los últimos años, ha aumentado la reacción contra el aborto y la autonomía reproductiva, impulsada por regímenes autoritarios e ideologías conservadoras. En 2023, CommonHealth realizó un estudio para identificar entidades clave antielección en India, examinar sus vínculos mundiales y analizar sus narrativas y estrategias para influir en la opinión pública y en las políticas por medio de plataformas mediáticas (impresas y sociales) en tres idiomas. Los principales hallazgos fueron que estas organizaciones están aprovechando las plataformas educativas y las redes sociales, cooptando campañas contra la discriminación de género y planteando sus argumentos con retórica moral y constitucional. Algunas de sus tácticas son la manipulación de la percepción pública y ganar confianza al combinar la ciencia, la religión y el apelar a las emociones. Para influenciar a jóvenes en particular y obtener apoyo contra la elección reproductiva, se utilizan líneas de ayuda en situaciones de crisis, extensión educativa, campañas de redes sociales, esfuerzos de movilización masiva e interacciones directas entre pares. Además, el estudio destaca la creciente influencia de redes internacionales en estas iniciativas. Las narrativas emergentes se centran cada vez más en entrelazar ideologías antiaborto e ideologías antielección más amplias, haciendo hincapié en los derechos fetales y en la santidad de la vida. Por ello, estas iniciativas representan una amenaza considerable para los derechos reproductivos y la autonomía al socavar el acceso a los servicios de aborto seguro y legal. Para contrarrestar este activismo antiaborto en evolución, es imperativo crear intervenciones y estrategias específicas para cada contexto. Este análisis procura contribuir a la comprensión matizada de los retos, haciendo un llamado a la colaboración interdisciplinaria entre las partes interesadas para salvaguardar y fortalecer la justicia reproductiva. Por consiguiente, repensar el aborto como una decisión que mejora la vida es esencial para promover los derechos reproductivos y la autonomía en medio de narrativas antielección que se intensifican.

## Introduction

### Background

Abortion rights have been a deeply divisive issue worldwide. The global landscape of abortion rights is shaped by a complex interplay of legal, social, and political factors that differ significantly across countries and regions, often influenced by the political climate in key nations. In recent years, there has been an intensified global backlash against sexual and reproductive autonomy, driven by the rise of authoritarianism and conservative socio-political ideologies.^1^ The 2022 reversal of *Roe v. Wade* by the United States (US) Supreme Court in its ruling in the *Dobbs v. Jackson Women's Health Organization case*^2^ serves as a stark reminder of the fragility of these rights, even in nations where they have been long established. Although global movements to secure and expand abortion rights had made considerable progress – with several countries decriminalising abortion and enacting laws to protect reproductive rights – these advancements have been met with vigorous opposition from anti-abortion movements. Following the overturning of *Roe v. Wade*, these movements have gained momentum worldwide, particularly in regions where conservative or religious sentiments are already strong. This decision has not only curtailed reproductive rights in the US but also emboldened anti-abortion forces across the globe.^3^

In many countries, well-funded, transnational networks share strategies, rhetoric, and resources to bolster anti-abortion movements.^4^ As a result, nations with historically progressive abortion laws are facing renewed challenges, while those with restrictive laws see an opportunity to further tighten access to reproductive healthcare. Certain self-proclaimed “pro-life” religious/faith-based organisations in India celebrated the US Supreme Court's ruling, viewing it as a victory that could inspire similar actions against abortion laws worldwide.^5^ This celebration highlighted a growing and deliberate opposition to abortion rights in India, despite it being one of the first countries to liberalise access, through the enactment of the Medical Termination of Pregnancy (MTP) Act in 1971.

### Historical trajectory of anti-abortion sentiment in India

The landscape of reproductive rights and choices across countries has consistently been shaped by the clash between liberal and conservative values, with debates on sexual morality often prompting state intervention.^6,7^ In India, opposition to abortion is not a new phenomenon. A decline in the Christian population in Kerala,* a Southern state, had sparked the initial debate as far back as 2007.^8^ However, the movement did not gain significant traction or become widely visible due to India’s complex socio-cultural and legal framework. Attitudes towards abortion in India have largely been influenced by economic aspirations, the state's goals of promoting smaller family sizes, and women's actual and perceived ability to make decisions about their reproductive health.^9^ Legal regulations and religious beliefs have played a comparatively smaller role, making a simple “pro-life versus pro-choice” binary almost impossible in such a multifaceted reality.

Globally, the legality of abortion in many countries, including the landmark *Roe v. Wade* ruling of 1973 in the US, the Causa Justa movement in Colombia (2022) and constitutional guarantees in France (2024), emerged from a broader context of rights movements. In contrast, the feminist movement in India has historically not significantly prioritised advocacy for reproductive rights and choices, particularly concerning abortion. The MTP Act, which was established over five decades ago in 1971, permitted the termination of pregnancy under a wide range of conditions by qualified professionals in approved facilities.^10^ This legislation was driven by concerns about maternal mortality and, more subtly, by worries about the rapidly growing population.^11,12^ There are structural barriers, including legal obstacles, inadequate public healthcare infrastructure, a shortage of non-judgmental abortion providers, and pervasive social stigma, that continue to disproportionately impede access to safe abortion care for vulnerable populations, particularly young people, marginalised gender identities, and lower socio-economic groups in the country.^13^ However, the 2021 amendment to the MTP Act, intended ostensibly to expand access to services and ensure dignity, autonomy, confidentiality, and justice for those seeking abortions, was also met with criticism on various fronts.^14^

Pro-choice activists criticised the amended law for continuing to rely on permissions and exceptions, thereby challenging a rights-based approach to healthcare. On the other hand, the government's attempt to introduce task sharing as per WHO guidelines for providers, aimed at improving access to abortion services, faced opposition from the medical community due to concerns about relaxing professional standards.^15^ The Catholic Church also opposed the amendments, invoking the deeply ingrained concept of Ahimsa – the ancient principle of nonviolence which applies to actions towards all living beings – in the Indian religious majority to garner broader support against the changes.^16^

The 2022 Supreme Court decision in the US, which overturned *Roe v. Wade* on the grounds that abortion is not a right deeply rooted in the nation’s history or tradition, was expected to bolster the global anti-abortion movement. In India, for example, on August 10, 2022, Catholic Charismatic Renewal Services and the Delhi Catholic Charismatic Service of Communion organised the “March of Life” protest against abortion. Held symbolically on the 51st anniversary of the MTP Act, this event was labelled a “Day of Mourning” for the millions of fetuses that had been aborted.^17^ Around 100 religious leaders and functionaries participated in the march, which concluded with participants expressing hope that their actions would inspire others to join the anti-abortion movement and ultimately lead to the repeal of the MTP Act.

The September 2022 landmark ruling of the Supreme Court of India,^18^ which recognised the rights of unmarried women, marital rape survivors, and others to exercise their reproductive autonomy, including access to safe and legal abortions, aggravated anti-choice groups. Catholic Church representatives responded by warning that the decision could disturb social equilibrium, undermine the moral compass of society, and lead to adverse demographic consequences.^19^

Since then, abortion opponents in India, led by the Catholic Church, have organised annual “March for Life” events across various cities. Over time, certain Hindu organisations also have begun promoting anti-abortion content on platforms like X and YouTube, portraying abortion as contrary to Hindu traditions.^20,21^ Recognising the threat these initiatives pose to sexual and reproductive health, rights, and justice, it is crucial to closely monitor and analyse emerging anti-abortion trends and narratives and develop informed counter-strategies to protect and advance these essential rights.

### Efforts at countering anti-abortion movement

The struggle for abortion rights is not confined to a specific region or country but is a global issue with profound local consequences. To effectively counter the challenges posed by anti-abortion movements and protect the progress made in reproductive rights, it is essential to understand both global and national dynamics. In the face of these global challenges, feminist activism has been instrumental in advancing sexual and reproductive health, rights, and justice (SRHRJ). Notable progress has been made through global South–South transnational activism, as seen in the Green Wave movement in Latin America^22^ and initiatives to dismantle colonial-era abortion laws in Africa.^23^ Building on this momentum, organisations in the Global South, including in India, Nigeria, Kenya, and Colombia, collaborated to examine the impact of the *Dobbs* judgment and develop evidence-based advocacy strategies for advancing SRHRJ.^24^ Their efforts aim to counteract the global impact of *Roe v. Wade*'s overturn. As anti-abortion movements increasingly utilise social media and digital platforms with vast reach to spread their message, mobilise supporters, and pressure governments to restrict or ban abortion, it is crucial for reproductive justice advocates to remain vigilant.

This paper is based on the findings of a study in India that examines the discourse generated by anti-choice entities, and the recurring themes, arguments, narratives, outreach strategies, and framing tactics they use to influence public opinion and policy. It also explores their public outreach strategies and mass mobilisation efforts.

## Methodology

### Objectives

A study was carried out to identify key anti-choice entities in India (and their overseas linkages), individuals, and groups actively engaged in disseminating messaging or content challenging reproductive autonomy and abortion rights of cis-women and gender-diverse people on media platforms/outlets, and analyse the discourse generated by them.

### Data collection methods

For data collection, the study used a curated list of code words, keywords, hashtags, and phrases (provided in Supplementary File 1) that are commonly found in anti-abortion discourse in English, Hindi, and Assamese, the languages familiar to the study team. This list was employed to search for anti-choice content using advanced search features on platforms such as Twitter/X and Google Social Search. Additionally, Google Alerts were set up for ongoing monitoring, providing notifications for newly published content containing these specific keywords. It tracked and analysed components including websites, affiliated blogs, and social media accounts (YouTube, Facebook, Twitter, and Instagram) of the organisations disseminating anti-abortion messaging listed in Supplementary File 2. Desk research on recent studies investigating anti-abortion activism in the US and the UK was also conducted to identify transnational parallels and influences within the anti-choice movement.

### Ethics approval

The study was formally approved by the Steering Committee and Project Advisory Committee of CommonHealth, who reviewed its objectives and methodology, and concluded that no additional institutional ethics approval was required. This decision reflected the fact that all material analysed was publicly available and had been disseminated by registered, public-facing organisations.

Although the data were collected from openly accessible online sources, including websites and social media platforms (YouTube, Facebook, X, and Instagram), the research team acknowledged that public availability alone is not sufficient to justify ethical use. The study was guided by the objective of generating new knowledge about an emerging discourse and movement in India that seeks to restrict reproductive autonomy.

Borrowing guidance from the Association of Internet Researchers (AoIR)^†^ on internet-mediated research, the study deliberately focused only on outputs created and disseminated by formal organisations, thereby avoiding the more complex ethical challenges associated with analysing the content of private individuals. No images or materials that could potentially identify individuals were used; the analysis was limited to institutional content.

Geographical information included in the study refers solely to organisations (e.g. their declared headquarters or stated regional focus) and is not connected to any personal or individual-level data.

The inclusion of direct quotations, images, and posts has been undertaken with careful consideration of national and international copyright laws, including the doctrine of fair use under Section 52 of the Indian Copyright Act, 1957, Section 107 of the US Copyright Act, and Section 30 of the UK Copyright, Designs and Patents Act, 1988. These provisions allow the reproduction of copyrighted material for purposes such as criticism, review, and academic analysis. All excerpts and images have been properly attributed to their original organisational sources.

### Timeframe

Data was collected over four months from September 2023 to December 2023. The content analysed, however, encompassed a five-year timeframe, ranging from 2018 to 2023.

### Analytical framework

Qualitative methods of conceptual and relational content analysis were undertaken to identify and break down anti-abortion data. The data comprised over 1000 Instagram posts, 19 tweets, 21 YouTube videos, 20 Facebook posts, and 7 websites. This analysis aimed to trace key opposition entities and explore their discourse-framing tactics, public outreach strategies, and mass mobilisation efforts. For conceptual content analysis, the presence and frequency of curated terms/concepts, code words, and phrases mentioned in File 1 were explored. For the relational analysis, the connections between these particular concepts were examined.^25^ The analysis also examined the demographic composition of target audiences, geographical presence, and operational timelines of these entities to gain insights into their influence.

Among the frequently occurring terms, content, concepts, and inter-concept relationships, certain dominant themes and narratives emerged. Thematic analysis was carried out to identify these narratives, recurring messages, and overarching trends shaping the discourse within the anti-choice landscape. For the purpose of this analysis, the terms “anti-choice entities” and “anti-abortion groups” or “anti-abortion organisations” are used interchangeably to refer to organisations and/or groups opposed to abortion rights on various grounds. This opposition was either explicit: through direct and clear disapproval or rejection of abortion via public statements, advocacy, or organised campaigns, or implicit: manifested through subtler or indirect actions, policies, or messaging that creates barriers to abortion access. The term “pro-life,” appropriated by these entities to claim the moral high ground, has been intentionally avoided to emphasise that being pro-choice does not equate to being “anti-life.”

### Study team

The analysis involved a team of four researchers. Two social scientists/researchers conducted the initial stages, including the curation of code words, the first level of content and relational analysis followed by thematic analysis. Subsequently, two senior researchers reviewed their findings on a sample basis (depending on their familiarity with the language in which data was available) focusing particularly on thematic and discourse analysis in the context of opposition to abortion.

## Findings: the evolving anti-abortion discourse in India

This section unpacks the findings of the study, focusing on the organisations, narratives, and strategies shaping this discourse.

The first section**, Unveiling the frontlines: Key anti-abortion forces in India,** identifies and examines the principal entities opposing abortion rights in India, categorising them into non-governmental organisations (NGOs) and faith-based organisations. It highlights their objectives, operational structures, and target demographics. The second section, **Emerging narratives in the anti-abortion discourse and movements,** explores the evolving themes in anti-abortion rhetoric, including the intersections with broader anti-choice ideologies, the emphasis on fetal rights, and moral and constitutional arguments used to challenge existing laws. The third section, **Tactical approaches,** explains the methods employed by these organisations to advance their agenda. This includes the use of visual imagery, manipulation of scientific data, and co-opting of feminist and human rights rhetoric to influence public perception. The final section, **Public engagement strategies,** outlines various approaches employed by anti-abortion organisations to engage with the public. These include operating crisis helplines, conducting educational outreach programmes leveraging social media campaigns, organising mass mobilisation efforts, and facilitating direct peer interactions.

### Unveiling the frontlines: key anti-abortion forces in India

An analysis of over 1000 monitored digital media entries identified eight key entities actively opposing abortion rights in India. These include four NGOs and four faith-based organisations. While the NGOs function independently or through regional networks, the faith-based organisations align with larger religious institutions. The NGOs include Life for All, The Saved Pearl Foundation, Janpragati, and Rescue Charitable Trust. These organisations have been active for more than a decade, with some, such as Life for All, extending their reach internationally. On the other hand, the faith-based organisations – Eva Pro-Life Movement, Youth United for Christ, Jesus Youth Pro-Life Movement, and Sant Shree Asharamji Ashram – are deeply rooted in religious networks. Three of these faith-based organisations are Catholic, while the fourth is a Hindu group.

All eight entities focus their anti-abortion messaging and outreach on women, youth, queer individuals, and transgender people, employing varied strategies to influence public opinion and policy.

#### NGOs

Life for All, an international NGO established in 2009 and headquartered in Tamil Nadu (one of India's more socially progressive states) claims to focus on defending the value of human life from conception to natural death. This organisation operates “Life Centres” for those experiencing crisis pregnancies,^‡^ facilitates adoptions, and provides counselling services through its initiative, *Pregnancy Helpline India*. Beyond opposing abortion, it claims to bolster the anti-abortion movement by training leaders and supporting pregnant persons through education, life skills, and medical aid. According to self-reported data published on its official website, the organisation has engaged with 134,749 individuals.^26^

The Saved Pearl Foundation, founded in 2015 and based in Mumbai, India's commercial capital, focuses on providing support to pregnant persons, particularly those facing crises stemming from financial hardship, sexual violence, abandonment, or abusive relationships. The organisation engages in raising awareness about crisis pregnancies and gender-based sex-selective abortions. It also conducts moral awareness workshops in educational institutions, promoting abstinence-focused sex education. According to the organisation’s self-reported data, its initiatives have reached over 10,200 adolescents and more than 2,200 women from low-income communities, in addition to reportedly “saving 33 babies.”^27^

JanPragati NGO, established in 2011, in Uttar Pradesh, India's most populous state, ostensibly dedicated to poverty alleviation through slum education and women's empowerment, also operates a crisis pregnancy centre. Through initiatives such as the “Sanctity of Life” project and the “Speak Life” campaign, the organisation promotes the belief that life begins at conception and emphasises the inherent value and rights of the fetus, while encouraging women experiencing “crisis pregnancies” to continue their pregnancies.^26,28^

Rescue (Rescue Charitable Trust) is an NGO established in 2011 and based in Karnataka – India's Silicon Valley – focused on preventing abortion following gender-biased sex-selection, which it terms “baby genocide.”^29^ The organisation also targets youth with moral awareness programmes in schools, linking abortion to irresponsible sexual behaviour.

#### Faith-based organisations

The Eva Pro-Life Movement serves as the official pro-life organisation of the Kalyan Eparchy, an Eastern Catholic eparchy established in 1988 under the Syro-Malabar Catholic Church, headquartered in Kalyan, Mumbai. The movement professes a commitment to defending the dignity of human life, particularly during vulnerable stages such as pregnancy and end-of-life. It operates Garbhadhrithi, a free counselling helpline that provides psychosocial support to those encountering unplanned pregnancies. Beyond its staunch opposition to abortion and its critique of the MTP Act, the organisation also rejects contraception, masturbation, same-sex relationships and assisted reproductive technologies such as in-vitro fertilisation (IVF), reflecting its broader ideological stance. It has also been an active participant in the “March for Life” public demonstrations demanding the repeal of the MTP Act in India since 2022.

Youth United for Christ, based in Mumbai, Maharashtra, is the official youth ministry of the Catholic Charismatic Renewal International Service (CHARIS) India, an affiliate of CHARIS International recognised by the Holy See. The organisation extends its influence through regional chapters in Goa, Vasai, Pune, Meerut, Chennai, and Darjeeling. Since 2022, it has actively mobilised public support for the repeal of the MTP Act of 1971, organising annual “March for Life” events that have drawn significant participation, including schoolchildren, teenagers, young adults, and gynaecologists. In addition to these public demonstrations, it also conducts training programmes and conferences specifically tailored for young people, focusing on promoting anti-choice ideologies.

The Jesus Youth Pro-Life Ministry, an international Catholic initiative recognised by the Holy See, operates a National Pro-life Ministry in India dedicated to “defending the gift of life from the moment of conception until natural death.”^30,31^ This ministry focuses on fostering youth engagement through the organisation of training courses, seminars, and pro-life exhibitions, aiming to instil anti-abortion ideologies among young people and adolescents. Additionally, the movement has played an active role in the “March for Life” public demonstrations since 2022, further solidifying its presence in anti-abortion advocacy efforts.

The Hindu organisation, Sant Shree Asharamji Ashram, headquartered in Gujarat, operates an extensive network comprising over 400 ashrams and 40 schools both within India and internationally. The organisation maintains a significant presence on social media platforms, including X (formerly Twitter) and YouTube, which it utilises to disseminate anti-abortion messaging. Its women’s wing, the Mahila Utthan Mandal, spearheads the “*Garbhapaat Mahapap Abhiyan*” (translated as Abortion is the Greatest Sin Campaign), which portrays abortion as fundamentally opposed to Hindu traditions. This campaign aims to instil good values from infancy and to address social issues such as abortion and Caesarean deliveries.^32^

Based on publicly available information, three of these entities have identifiable overseas collaborations and partnerships, though details about funding connections remain unclear. Life for All NGO has collaborated closely with a US-based anti-abortion organisation, whose Founder and President served as the Chief of the 8th National Pro-Life Summit held in Kolkata in August 2023.^33^

Youth United for Christ's “Flesh and Bones” Pro-Life Formation Training Course featured several international pro-life influencers as speakers. Additionally, digital media content, including videos and social media posts from Live Action Org – a US-based non-profit known for its anti-abortion activism – is frequently amplified by organisations such as Eva Pro-Life Movement and Jesus Youth Pro-Life Ministry.

### Emerging narratives in the anti-abortion discourse and movements

Emerging narratives within the anti-abortion and anti-choice discourse in India increasingly coalesce around three central themes: the intersection of anti-abortion and broader anti-choice narratives, the prioritisation of fetal rights and the sanctity of life, and the strategic deployment of moral and constitutional arguments.

#### Anti-abortion and anti-choice narratives

Opposition to abortion in India is deeply embedded within a broader anti-choice movement that condemns various sexual and reproductive choices, including same-sex relationships, premarital and casual sex, and the use of contraception. These organisations primarily advocate for traditional values such as abstinence and the sanctity of marriage and motherhood. To advance this narrative, they often employ stigmatising language, equating premarital sex with sin and homosexuality with “unnaturalness.” Additionally, they distort scientific evidence to support their claims. For instance, Mahila Utthan Mandal's website states claims that abortion can lead to “two and a half times higher risk of cervical cancer and 50 percent higher risk of ovarian cancer … decreased morale, headache, irritability, suicidal thoughts and increased mental stress.”^34^ Eva Pro-Life’s website for its crisis pregnancy helpline states that “all women, especially young teenagers, are at risk for cervical damage during abortion” (attributed to Schulz KF, Grimes DA, Cates W Jr., Measures to prevent cervical injury during suction curettage abortion, *The Lancet*, 28 May 1983). The study does not make this claim, however. It rather focuses on identifying clinical practices that reduce cervical injury in early suction curettage procedures.^35^

#### Fetal rights and the “Sanctity of life” arguments

A significant aspect of the anti-abortion narrative is the focus on fetal rights, rooted in the belief that life begins at conception. This perspective frames the fetus as a fully human entity with legal and moral rights, deserving of protection. The argument for fetal rights is often used to challenge and restrict access to abortion services. To bolster this narrative, these anti-abortion organisations use emotionally charged language and imagery. For example, an Instagram post by Eva Pro-Life Movement says, “The right to life is inherent and universal, transcending all boundaries of location. Whether inside the womb or outside, every human being possesses an equal and unalienable right to life.”^36^

#### Moral and constitutional arguments

Anti-abortion organisations argue that the MTP Act infringes on the constitutional rights of the “unborn human” by permitting abortion. By framing abortion as a violation of alleged fetal rights, they aim to build a moral and ethical case against the Act. The annual “March for Life” rallies, held on August 10th to coincide with the Act's anniversary, serve as a significant demonstration of the growing public activism against it. Additionally, young anti-abortion activists are using social media platforms such as Instagram to highlight perceived constitutional flaws in the Act, further fuelling opposition to it. It quotes the basic and inalienable right to life enshrined in Article 21 of the Indian Constitution^37^ while an X/Twitter post by Mahila Utthan Mandal states that while the murder of a human being attracts Section 302 of the Indian Panel Code, “abortion or foeticide” is equivalent to killing a sage,^38^ for which the punishment has to be faced in this world of the other world, thus amalgamating legal and moral arguments.

### Tactical approaches

Anti-abortion organisations use sophisticated and strategic tactics, including spreading scientific and medical misinformation, co-opting gender-based campaigns, and employing fetal imagery and personhood rhetoric to manipulate public opinion. They exploit gender issues and adopt human rights and pro-women narratives, glorify motherhood, and use religious justifications to strengthen their influence.

#### Use of fetal imagery and personhood rhetoric

Over half of the analysed digital content featured graphic imagery of developing or aborted fetuses, either real or simulated, paired with emotionally charged language like “murder” and “baby” to highlight fetuses’ potential humanity. For instance, Jesus Youth Pro-Life Ministry’s Instagram posts argue that embryos are functional human beings from their earliest stages deserving recognition and protection,^39,40^ while Eva Pro-Life Movement emphasises the visible facial features, rapidly developing brain, and distinct blood type of a seven-week-old fetus (see Supplementary File 3, Figure S1).

#### Adoption of human rights narrative

Faith-based organisations such as Eva Pro-Life Movement frame their anti-abortion stance within a human rights context arguing that life begins at conception and portraying the fetus as a rights-bearing entity. Both Jesus Youth Pro-Life Ministry^41^ and Eva Pro-Life Movement^42^ also use disability rights rhetoric to assert the values of all lives, aligning their stance with the principles of inclusivity and equality from the disability rights movement, to bolster their position and appeal to a broader audience.

#### Fabrication of scientific information

A discernible trend among anti-abortion organisations involves the increasing adoption of an ostensibly science-based information. They selectively cite research on abortion to enhance the credibility of and lend weight to their arguments. They often misrepresent scientific studies focusing on fetal development, the psychological impact on those seeking abortions, and alleged long-term health risks associated with abortion as shown in Figure S2 (see Supplementary File 3). This tactic creates a facade of scientific legitimacy, allowing these entities to present their arguments as evidence-based and credible.

#### Spread of medical misinformation

In the ongoing battle for reproductive rights, a key tactic is the deliberate dissemination of medical misinformation. For instance, Mahila Utthan Mandal’s website falsely claims that abortion increases risk for breast cancer, reduction in fertility, and potential disabilities in children born thereafter. They even misleadingly connect abortion to various health issues, including menstrual disorders and cancers.^43^ Using medical professionals as spokespersons adds unwarranted credibility to these claims.

#### Distortion of abortion data

Research is selectively quoted or distorted to amplify perceived dangers associated with abortion. A prominent example is the misuse of data from the 2015 study *The Incidence of Abortion and Unintended Pregnancy in India*, published in The Lancet Global Health, to reinforce their anti-abortion stance, potentially skewing the study's findings and the broader context of reproductive health in the region.^44^ On January 24, 2019, *The Hindu*, a widely respected newspaper, while reporting on a silent march organised by “Life For All” on National Girl Child Day, included a quote from the organisation's founder, “*As per a study published in the Lancet Medical Journal on the incidence of abortion and unintended pregnancy, 15.6 million abortions were reported in India in 2015. Every second or third pregnancy in the country ends in abortion, a major reason for which is sex-selective abortion which we are fighting to end*” – a prime example of statistical misinterpretation.^45^ While the number of abortions is accurately cited, the conclusion drawn is deeply flawed, ignoring that most were first-trimester and medication-based. Also the original study made no such claims. This misrepresentation shifts focus from the need for accessible, safe abortion services to a misleading narrative of gender-biased sex-selective abortions.

#### Exploitation of gender issues

Gender-focused campaigns like Pre-Conception and Pre-Natal Diagnostic Techniques (PCPNDT) Act and *Beti Bachao, Beti Padhao* (“Save the Girl Child, Educate the Girl Child”) are exploited to stigmatise abortion. While these campaigns were originally intended to address skewed sex ratios and combat son preference, these organisations misuse them to undermine abortion rights. By invoking terms like “Save the Girl Child” and *kanya bhrunhatya* with emotional language and imagery, they create a façade of supporting women’s rights. This tactic is certainly not a case of inadvertent misinterpretation; rather, it is a calculated strategy designed to align with the government’s stance, garner broader appeal, and push an anti-abortion agenda that extends well beyond the issue of gender-biased sex-selective abortions.^46–48^

#### Appropriation of a pro-women stance

Similarly, feminist rhetoric is appropriated to justify limiting abortion rights, framing it as essential for combating gender-based discrimination and protecting female lives, as shown in Figure S3 (see Supplementary File 3). Crisis pregnancy support groups of these organisations bolster this narrative by claiming they empower women who feel pressured into abortion due to external factors.^49,50^ They assert that true feminism values the rights of unborn females, respects the dignity of motherhood, and empowers women without forcing them to choose between careers and family. These organisations also challenge the notion that women must choose between a career and motherhood, or that women in the womb or those with disabilities are less deserving of life, advancing their anti-abortion stance under the guise of promoting gender equity and inclusivity.

#### Glorification of motherhood

Positive storytelling is strategically employed by featuring video testimonials from couples who continued with their pregnancies despite unplanned circumstances or a diagnosis of fetal disability. Rather than portraying abortion as a choice, these stories focus on the fulfilment and gratitude felt by individuals who chose to give birth, framing carrying a pregnancy to term as a positive and rewarding decision, even in difficult situations.^51^

#### Religious justification

Religious-/faith-based organisations opposing abortion use sacred texts from the Bible and Hindu scriptures to underscore the sanctity of life from conception and to condemn abortion as “killing” or “murder.” Verses like Jeremiah 1:5 (“*Before I formed you in the womb, I knew you*”) and Parashara Smriti 4:20 (“*Abortion incurs a sin twice that of killing a Brahmana and has no atonement*”) are frequently cited in their materials. These organisations emphasise the vulnerability of the unborn child, who cannot protect itself or cry out for help, reinforcing their moral and religious arguments against abortion.^52,53^

### Public engagement strategies

Anti-abortion organisations employ a range of public engagement strategies, including crisis helplines, educational outreach, social media campaigns, mass mobilisation efforts, and direct peer interactions, all designed to influence public opinion and advance their anti-choice agenda.

#### Crisis helplines

Organisations such as Life for All, JanPragati, The Saved Pearl Foundation, and Eva Pro-Life Movement run free, confidential helplines for those facing unplanned pregnancies. While offering counselling via one-to-one calls or chats, they primarily push adoption or parenting as the only options, connecting callers to Crisis Pregnancy Centres that reinforce this narrative through links with the Child Welfare Committee (CWC) and Central Adoption Resource Authority (CARA).

These helplines employ disinformation tactics, as depicted in Figure S4 (see Supplementary File 3), emphasising negative physical and emotional consequences associated with abortion procedures to dissuade clients from choosing abortion, thereby restricting their reproductive choices. Life for All’s “Pregnancy Helpline India” controversially promotes so-called “abortion pill reversal” within 72 hours of taking abortion medication pills, a procedure that is medically unproven and highly contested.^54^

#### Educational outreach

Organisations like Mahila Utthan Mandal, CHARIS-India, Jesus Youth, Life For All, and Rescue NGO run educational programmes that subtly promote anti-abortion ideologies, for example, a pro-life exhibition at a nursing college in Karnataka displaying silicon fetuses and anti-abortion posters.^§^ Mahila Utthan Mandal hosts “Stop Abortion” seminars, while CHARIS-India and Jesus Youth Pro-Life Ministry spread anti-choice messages through courses. As of December 2023, Life For All claims to have reached over 130,000 participants across 700 seminars, and Rescue NGO's campaign has engaged 186,000 students. These programmes often feature presentations by experts on the “detrimental effects of abortion” and use pro-life exhibits with fetal imagery to influence perceptions. Life for All also offers counselling certification programmes focused on fetal development, adoption, and “sexual integrity” to shape views on abortion and reproductive rights and draw them into the anti-abortion movement.

#### Social media campaigns

On August 10th each year, anti-abortion organisations leverage social media to mark the anniversary of the MTP Act, as a “Black Day.” This annual event is designed to cast a shadow over the legislation that enables safe and legal abortions in India. In August 2021, CHARIS India’s Youth United for Christ led a Digital Blackout Campaign, urging people to use black posters as WhatsApp display pictures in protest. Such campaigns harness the power of social media, as shown in Figure S5 (see Supplementary File 3), particularly platforms like WhatsApp, to rapidly spread their message and create an illusion of widespread opposition to abortion.

#### Memorial services

Some organisations organise memorial services to honour what they describe as “unborn children lost to abortion.”^55^ Jesus Youth Pro-Life Ministry's “Choose Life Campaign” in Bangalore, held a 40-day intercession and awareness effort in February 2021, spreading the “Gospel of Life” and praying for those they see as victims of abortion. Similarly, Eva Pro-Life Movement hosted a “Cemetery of the Unborn” event in June 2023, where young participants prayed for both unborn babies and individuals involved in abortions, symbolically mourning the lives they claimed were lost.

#### Public outreach

Youth volunteers from organisations like Jesus Youth Pro-Life Ministry and Rescue NGO actively engage with peers in public spaces, such as parks, to sway opinions on abortion. They often use emotional appeals and fetal imagery to spark conversations and elicit strong emotional responses, aiming to convert people to a pro-life perspective.^56^

#### Youth engagement

Young people are being recruited into the anti-choice movement through educational outreach in schools and colleges, leading to a new generation of activists opposed to abortion. Events like the “March for Life” rallies energise these youth, who also leverage social media to spread their message, creating visually compelling and engaging content on platforms like Instagram. For example, Youth For Christ’s main Instagram account and regional chapters have a combined following of around 13,200 as of December 20, 2023.

#### Mass mobilisation

These organisations also engage in public activism through silent marches and rallies, often timed around significant dates like National Girl Child Day and the anniversary of the MTP Act. Life For All organised a silent rally on January 24, 2019, in Coimbatore to mourn girls lost to sex-selective abortions. CHARIS India, supported by Pro-Life Global, has been organising annual “March for Life” events since 2022 to campaign for the repeal of the MTP Act. These marches, held in cities like New Delhi, Pune, and Thrissur, draw diverse crowds, including young people, children, anti-abortion gynaecologists, lawyers/judges and other professionals. Eva Pro-Life Movement held a “Walk for Life” in December 2023 in Kalyan City, Maharashtra, featuring exhibitions with fetal imagery to further their cause.^57^

## Discussion and conclusion

Media actions by anti-choice entities in India stigmatise abortion, spread misleading information, and promote traditional roles to reinforce restrictive gender norms. Their efforts often lead to portrayal of service providers as unethical, divert attention from legal abortion rights, and exert pressure on policymakers by shaping public opinion. The anti-abortion movement thus poses a significant threat to reproductive rights and autonomy and undermines access to safe and legal abortion services. This can result in an increase in unsafe abortions, which carry severe health risks and can lead to adverse, and even fatal, outcomes for abortion seekers.

Key findings indicate that the public engagement strategies of these entities manipulate abortion seekers through selective options, disinformation, and unproven medical procedures. Emotional appeals and mass mobilisation aim to shift public attitudes toward an anti-choice stance, exposing a calculated effort to undermine reproductive autonomy. The use of graphic imagery, religious objections, and distorted scientific facts are meant to instil fear and uncertainty in abortion seekers. They twist human rights and equality arguments to construct rights-based claims and “pro-women” narratives that serve their anti-abortion agendas, often cloaking religious beliefs in secular and constitutional rhetoric. Similar rhetoric is observed among anti-abortion activists in the UK where a common tactic is the use of seemingly “secular” frames to promote anti-abortion arguments, even though the opposition is primarily rooted in religious beliefs, to broaden the appeal of these arguments.^58^ Further, crisis pregnancy centres in India, like those in the US, discourage abortions, exclusively promoting parenthood and adoption as the alternatives while spreading pseudo-scientific claims about abortion's negative health effects. These centres often operate without regulation and spread misleading or inaccurate information.^59^

Faith-based organisations and NGOs drive India's anti-abortion movement, mirroring trends in Africa where faith-based organisations play a pivotal role in shaping restrictive reproductive health policies. In Uganda and Kenya, cultural and religious values often shape policies. First Ladies play key roles; for example, Kenya's First Lady launched the Citizen Go-drafted National Family Protection Policy without public consultation, and Uganda's First Lady backed the Geneva Consensus Declaration – an anti-abortion joint statement – despite its lack of legal or policy authority.^60^

Activities of anti-choice entities in India are not isolated, they reflect a global trend, adapting tactics from the US and UK. While direct international ties are not always evident, collaborations with Europe and US-based anti-abortion influencers are growing, focusing on youth education and digital media resources from US-based “pro-life” organisations. Strategies like fetus-centric rhetoric emphasising “life from conception,” similar to the USA's “heartbeat” bills (e.g. Georgia HB 481 of 2019),^61^ are localised to promote fetal personhood, and thereby granting the fetus legal and moral protections. Alarmingly, judges and lawyers in India are also increasingly bringing up the fetus as a prominent entity in many abortion related cases, despite the absence of any legal recognition.^62,63^ The narrative of fetal interests needs to be carefully analysed by legal and constitutional ecosystems, including whether these are even legitimate issues for the State to pursue. Examples of recent law changes such as decriminalisation of abortion in 2021 in South Korea and on all grounds up to week 24 of pregnancy in 2022 in Columbia show how the narrative moved beyond the centrality of fetal interests in ensuring better access to abortion.^64,65^ These examples underscore the importance of examining transnational parallels to craft effective counter-strategies. By contrast, current trends in India have the potential to reshape the reproductive rights narrative in ways that may restrict access to abortion.

### Strengths and limitations

While the study undertaken does explore and analyse anti-abortion campaigns at the community, media, legal, policy and service delivery, and political levels, this paper limits itself to findings of media monitoring, i.e. the output of organisations, individuals and/or groups actively engaged in anti-abortion activities through media. The data collection was limited to keywords in three languages. Given the linguistic diversity in India, the scope of the findings therefore has limited generalisability.

Due to restrictions imposed by platforms such as Twitter/X on open access to their Application Programming Interfaces (APIs), data retrievals from these platforms were also limited to an extent. Tracking print media was challenging due to the limited searchability caused by the lack of digital archives for some of the older newspapers and, in some cases, the requirement for subscriptions, for which resources were not available.

The team also acknowledges that the availability and accessibility of data, particularly from more covert or less prominent anti-abortion groups and in other languages may have been missed as a result. Reliance on publicly available digital content may have resulted in an incomplete picture of the activities and influence of these entities.

Finally, the paper provides a snapshot based on the most recent and available data that may not fully capture long-term trends or shifts in the anti-abortion landscape.

However, the study adds value by shedding light on India’s unique socio-political landscape and highlights the growing challenges to reproductive autonomy. There is evolving evidence of increased influencing of local ecosystems towards restricting abortion, including a 2021 report by the European Parliamentary Forum for Sexual and Reproductive Rights which cites expenditure of USD 83 million in Europe by just 10 US-based entities – and this might be just the tip of the iceberg.^66^ The findings of this study also contribute to the global discourse by underlining how transnational anti-abortion movements are not only exporting rhetoric but also adapting local cultural narratives to reinforce their agendas.

Navigating this intricate landscape of evolving anti-abortion activism in India requires not only understanding the interplay of global and national factors shaping the anti-abortion discourse but also the urgent need for context-specific interventions, counter-strategies, and initiatives aimed at protecting reproductive autonomy and addressing the widespread stigma surrounding abortion. The study aims to foreground the urgent need for sexual and reproductive health and rights (SRHR) advocates, policymakers, and civil society to unite in countering anti-choice activism and advancing evidence-based strategies. It is imperative for the pro-choice movement to be vigilant in picking up warning signs of eroding rights-based narratives and proactively protect and advance rights-affirming discourses.

SRHR advocates in low- and middle-income countries must counter donor-funded anti-abortion activities that promote restrictive policies. By building localised, evidence-based counter-narratives, supporting community movements, and fostering international collaborations, they can challenge transnational anti-choice agendas and redirect advocacy to focus on rights and evidence, weakening restrictive ideologies as highlighted in global analyses of donor impacts on reproductive health policies.^67^

Additionally, recent shifts in leadership in the US offer a complex landscape for anti-abortion forces. SRHR advocates must therefore remain vigilant, leveraging these leadership changes as opportunities to highlight the adverse global consequences of restrictive US funding policies, such as the Global Gag Rule and recent Executive Orders, while advocating for increased support for comprehensive SRHR initiatives.^68^

Thus, through this paper, the authors aim to contribute to a more nuanced understanding of the challenges to reproductive autonomy and abortion rights in India and beyond to inform the need for evidence-based strategies that not only counter the impacts of anti-choice activism but also safeguard and strengthen the principles of reproductive justice.

## Supplementary Material

Supplementary File 3. Figs S1-S5

Supplementary File 1. Curated List of Codes, Keywords, Hashtags and Phrases.

Supplementary File 2. List of Anti-Choice Organisations.

## Footnotes

* One of three major religious communities in the state.

† AS Franzke, S Aline, A Bechmann, et al and the Association of Internet Researchers (2020). Internet Research: Ethical Guidelines 3.0. https://aoir.org/reports/ethics3.pdf.

‡ Unplanned pregnancies that are accompanied by adverse circumstances, involving health risks and/or financial, social, and relational difficulties.

§ Image available at https://www.instagram.com/p/C0crDb4yX6W/ by *jyprolife(*2023); Instagram.
