# Supplementary material for: Challenging choice: a media study of anti-abortion movements in India
Source: Sex Reprod Health Matters. 2026 Apr 15;33(1):2653891. doi: 10.1080/26410397.2026.2653891 (PMC13173564; doi:10.1080/26410397.2026.2653891)
Supplement: Supplementary File 3. Figs S1-S5 [file ZRHM_A_2653891_SM0739.docx]

**Supplementary File 3**

**List of Figures**

**Figure S1**

**Use of foetal imagery and personhood rhetoric**


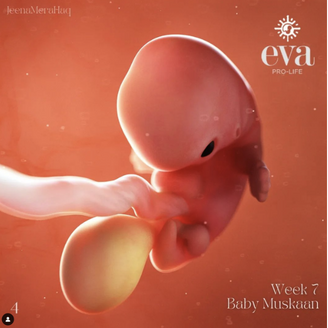


“WOW, we're at week 7! Baby Muskaan’s facial features are visible, including her mouth and tongue. The eyelids have begun to form. The major muscle systems have developed. She has her own blood type. 1,00,000 new brain cells are forming every minute. Her heart is beating 140-150 beats per minute—almost twice as fast as mom’s. Baby Muskaan even responds to tickling! At week 7, she is about an inch long [all stretched out] and weighs only 7 grams. Status: HUMAN.”

***Reference***: Image by Eva Pro-Life (2022); Instagram; reproduced under fair use (Section 52, Indian Copyright Act, 1957; Section 107 of the U.S. Copyright Act; Copyright, Designs and Patents Act 1988, Section 30).

**Figure S2**

**Fabrication of scientific information**
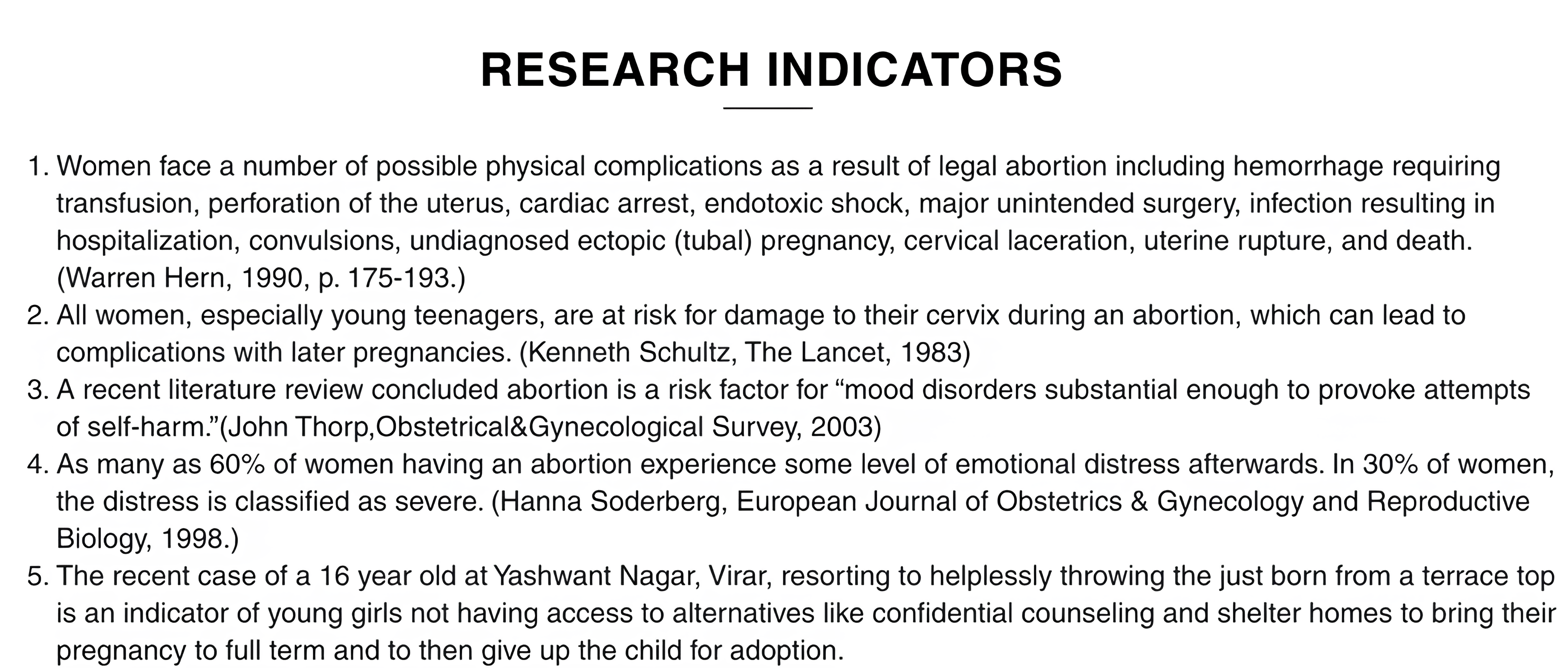


***Reference***: Image by Garbhdhriti Crisis Pregnancy Cell (2024); EVA Prolife Movement; reproduced under fair use (Section 52, Indian Copyright Act, 1957; Section 107 of the U.S. Copyright Act; Copyright, Designs and Patents Act 1988, Section 30).

**Figure S3**

**Appropriation of a pro-women stance**


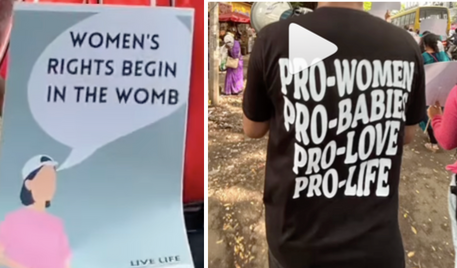


***Source***: National March for Life, India (2023); Instagram; reproduced under fair use (Section 52, Indian Copyright Act, 1957; Section 107 of the U.S. Copyright Act; Copyright, Designs and Patents Act 1988, Section 30).

**Figure S4**

**Disinformation by crisis helplines**


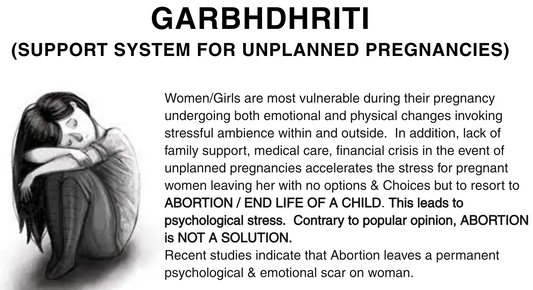


***Reference***: Image by Garbhdhriti Crisis Pregnancy Cell (2024); EVA Prolife Movement; reproduced under fair use (Section 52, Indian Copyright Act, 1957; Section 107 of the U.S. Copyright Act; Copyright, Designs and Patents Act 1988, Section 30).

**Figure S5**

**Social media campaigns**


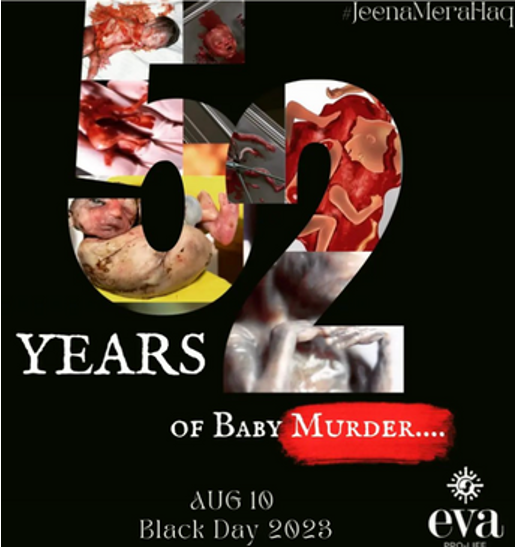


***Reference***: Image by Eva Pro-Life (2023); Instagram; reproduced under fair use (Section 52, Indian Copyright Act, 1957; Section 107 of the U.S. Copyright Act; Copyright, Designs and Patents Act 1988, Section 30).
