# Supplementary material for: Challenging choice: a media study of anti-abortion movements in India
Source: Sex Reprod Health Matters. 2026 Apr 15;33(1):2653891. doi: 10.1080/26410397.2026.2653891 (PMC13173564; doi:10.1080/26410397.2026.2653891)
Supplement: Supplementary File 1. Curated List of Codes, Keywords, Hashtags and Phrases. [file ZRHM_A_2653891_SM0738.docx]

**Supplementary File 1**

**Curated List of Codes, Keywords, Hashtags and Phrases**

**English**

#prolife

#prolifeindia

#marchforlifeindia

#antiabortion

“abortion + sin”

“abortion +murder”

“MTP 1971”

Abortion

MTP

Sex selection

Foeticide

Termination of pregnancy

Killing of innocent in the womb

Abort the fetus

Unwanted pregnancy

Pro-life

Pro-choice

March for life

Induced abortion

Foetal rights

**Hindi**

Garbhpat (गर्भपात)

Bhrun hatya (भ्रुण हत्या)

Garbh (गर्भ)

Pet girana/Pet girna (पेट गिराना/पेट गिरना)

Killing of unborn child (अजन्मे बच्चे की हत्या)

Kanya bhrun hatya (कन्या भ्रुण हत्या)

Bhrunnash (भ्रुणनाश)

Garbhsamapan (गर्भसमापन)

Unchaha garbh (अनचाहा गर्भ)

Medical Termination of Pregnancy Act (गर्भ के चिकित्सीय समापन अधिनियम)

Garbh girana (गर्भ गिराना)

Bachcha girana (बच्चा गिराना)

Kanooni Garbhsamapan (कानूनी गर्भसमापन)

गर्भपात+महापाप

**Assamese**

Gorbhopat (গভপাত)

Konya Bhrun Hotya (কনা ভ্ৰুণ হতা)

Bhrun Hotya (ভ্ৰুণ হতা)

Prorusito Gorbhopat (প্ৰৰুচিটো গভপাত)

Onirapod gorbhopat (অিনৰাপদ গভ পাত)]

Obanchito gorbhodharon (ওবাঞ্চিটো গভ ধাৰণ)

Lingo vittik gorbhopat (লিংগো ভিটিক গভপাত)

“Wash kori diya” (ৱাছ কৰা)

Gorbhowoti (গভৱতী)

Gorbhodharon (গভধাৰণ)
