# Supplementary material for: Challenging choice: a media study of anti-abortion movements in India
Source: Sex Reprod Health Matters. 2026 Apr 15;33(1):2653891. doi: 10.1080/26410397.2026.2653891 (PMC13173564; doi:10.1080/26410397.2026.2653891)
Supplement: Supplementary File 2. List of Anti-Choice Organisations. [file ZRHM_A_2653891_SM0737.docx]

**Supplementary File 2**

**List of Anti-Choice Organisations**

**Non-Government Organisations**

**1. Life For All**

**2. The Saved Pearl Foundation**

**3. Janpragati**

**4. Rescue Charitable Trust**

**Faith-based organisations**

**1. Eva Pro Life**

**2. Youth United for Christ (YU4C)**

**3. Jesus Youth (JY) Pro-Life Movement**

**4. Sant Shree Asharamji Ashram**
